# Variabilidade da Pressão Arterial em Única Visita e Risco Cardiovascular em Participantes do ELSA-Brasil

**DOI:** 10.36660/abc.20210804

**Published:** 2022-08-24

**Authors:** André Sant’Anna Zarife, Helena Fraga-Maia, José Geraldo Mill, Paulo Lotufo, Rosane Harter Griep, Maria de Jesus Mendes da Fonseca, Luciara Leite Brito, Maria da Conceição Almeida, Roque Aras, Sheila Maria Alvim Matos

**Affiliations:** 1 Universidade Federal da Bahia Salvador BA Brasil Universidade Federal da Bahia – UFBA, Salvador , BA – Brasil; 2 Hospital Geral Roberto Santos Salvador BA Brasil Hospital Geral Roberto Santos , Salvador , BA – Brasil; 3 Universidade do Estado da Bahia Salvador BA Brasil Universidade do Estado da Bahia – UNEB, Salvador , BA – Brasil; 4 Universidade Federal do Espírito Santo Centro de Ciências da Saúde Vitoria ES Brasil Universidade Federal do Espírito Santo – Centro de Ciências da Saúde , Vitoria , ES – Brasil; 5 Universidade de São Paulo São Paulo SP Brasil Universidade de São Paulo , São Paulo , SP – Brasil; 6 Fundação Oswaldo Cruz Rio de Janeiro RJ Brasil Fundação Oswaldo Cruz , Rio de Janeiro , RJ – Brasil; 7 Centro de Pesquisas Gonçalo Moniz Salvador BA Brasil Centro de Pesquisas Gonçalo Moniz , Salvador , BA – Brasil

**Keywords:** Pressão arterial, Fatores de Risco de Doenças Cardíacas, Hipertensão

## Abstract

**Fundamento:**

A variabilidade da pressão arterial (VPA) tem valor prognóstico para desfechos cardiovasculares fatais e não fatais.

**Objetivos:**

Este estudo teve como objetivo avaliar a associação entre a VPA em uma única visita e o risco cardiovascular em participantes do Estudo Longitudinal de Saúde do Adulto (ELSA-Brasil).

**Métodos:**

O presente estudo transversal foi conduzido com dados basais (2008-2010) de 14.357 participantes do ELSA-Brasil, sem história de doença cardiovascular. A VPA foi quantificada pelo coeficiente de variação de três medidas padronizadas da pressão arterial sistólica (PAS) realizadas com um oscilômetro. Medidas antropométricas e exames laboratoriais também foram realizados. O risco cardiovascular foi avaliado pelo estimador de risco de doença cardiovascular aterosclerótica (ASCVD), e se empregou a análise de regressão logística multivariada com nível de significância de 5%.

**Resultados:**

Um risco cardiovascular significativamente maior foi determinado por uma VPA elevada para ambos os sexos. Uma prevalência significativamente maior de alto risco foi observada mais em homens que em mulheres em todos os quartis, com a maior diferença observada no quarto quartil de variabilidade (48,3% vs. 17,1%). Comparações entre quartis por sexo revelaram um risco significativamente mais alto para homens no terceiro (OR=1,20; IC95%: 1,02 - 1,40) e no quarto quartis OR=1,46; IC95%: 1,25 -1,71), e para mulheres no quarto quartil (OR=1,27; IC95%: 1,03 - 1,57).

**Conclusão:**

Análises de dados basais de participantes do ELSA-Brasil revelaram que a variabilidade da pressão arterial se associou com risco cardiovascular aumentado, especialmente nos homens.

## Introdução

A hipertensão é considerada o principal fator de risco para doenças cardiovasculares, que vêm apresentando uma crescente mortalidade global nos últimos anos. ^1 - 3^ No Brasil, estima-se que a hipertensão arterial afeta aproximadamente 36 milhões de brasileiros, com uma prevalência entre 21,4% e 35,8% segundo estudos epidemiológicos. ^4 - 7^

Apesar de a hipertensão arterial ser considerada um fator de risco importante para acidente vascular cerebral e infarto agudo do miocárdio (IAM), evidências sugerem que a elevação na pressão arterial não é o único fator fisiopatológico relevante envolvido em eventos cardiovasculares. ^8 , 9^ Vários estudos demonstraram a importância da variabilidade da pressão arterial (VPA) na associação entre a hipertensão arterial e o risco cardiovascular. ^10 - 24^ A VPA é um fenômeno complexo, no qual flutuações da pressão arterial podem ser influenciadas pelo ambiente, comportamento, atividade do sistema nervoso central, e atividade hormonal de um indivíduo, entre outros fatores.

A VPA é avaliada batimento a batimento através de medidas intra-arteriais da pressão arterial, por médicos em ambiente clínico, ou utilizando-se um aparelho de Monitorização Ambulatorial da Pressão Arterial (MAPA), ou por monitorização residencial da pressão arterial (MRPA) em intervalos muito curtos, curtos, intermediários ou longos. ^25 , 26^ A VPA em curto prazo (24 horas), médio prazo (≥ 2 dias), e longo prazo (intervalos semanais, mensais, ou anuais) está associada com risco cardiovascular elevado, ^12 , 13^ hipertrofia ventricular esquerda, ^14 , 15^ espessura da íntima-média da carótida aumentada, ^16 , 17^ insuficiência renal crônica, ^18 , 19^ e eventos cardiovasculares fatais e não fatais. ^20 - 22^

Alguns estudos demonstraram que a VPA em uma mesma visita pode estar positivamente associada com risco de acidente vascular cerebral e doença cardiovascular, ^27 , 28^ enquanto outros não encontraram associações com desfechos cardiovasculares ou mortalidade total. ^29 , 30^ Dados previamente publicados do estudo do tipo coorte “Estudo Longitudinal de Saúde do Adulto” (ELSA-Brasil) relataram uma associação entre a VPA em uma mesma visita e a espessura da íntima-média carotídea. ^17^ Apesar do conhecimento acumulado na literatura, ainda não se pode concluir que a VPA representa um fator de risco independente que deveria ser modulado e controlado por tratamento anti-hipertensivo, ou simplesmente um marcador que acompanha a pressão arterial elevada. ^26^ Assim, o presente estudo teve como objetivo estabelecer associações entre VPA em uma única visita e risco cardiovascular entre participantes da linha de base do ELSA-Brasil. ^31^

## Métodos

### Delineamento do estudo

Este foi um estudo transversal conduzido com dados basais (2008-2010) do estudo ELSA-Brasil. O ELSA-Brasil é um estudo do tipo coorte iniciado em 2008, envolvendo funcionários públicos de instituições de educação superior e de pesquisa localizadas em seis capitais brasileiras (Salvador, Vitória, Belo Horizonte, Porto Alegre, São Paulo e Rio de Janeiro), com o objetivo de investigar a incidência e a progressão de doenças cardiovasculares e diabetes, bem como fatores biológicos, ambientais, psicológicos e sociais associados. A coleta de dados basais consistiu em entrevistas, medidas antropométricas, exames clínicos e coleta de amostras biológicas. Os participantes são contatados por telefone anualmente para registro de eventos de saúde, e a cada quatro anos são chamados para novas entrevistas, e avaliação do estado de saúde e desfechos. ^32^

### População

Foram incluídos servidores públicos ativos e aposentados de seis instituições, com idade entre 35 e 74 anos. Foram considerados não elegíveis indivíduos com deficiência cognitiva ou de comunicação séria, indivíduos com pretensão de se aposentarem em breve, ou que se aposentaram e se mudaram para uma residência distante de seus centros de pesquisa locais. Mulheres grávidas e mulheres que deram à luz menos de quatro meses antes da visita basal foram também consideradas não elegíveis. Do total de 15.105 participantes do período basal do ELSA-Brasil, excluímos 749 (5,0%) que relataram história de acidente vascular cerebral, infarto do miocárdio, revascularização ou insuficiência cardíaca. Assim, a amostra de nosso estudo foi composta de 14.357 indivíduos. Todos os participantes assinaram um termo de consentimento, e o protocolo do estudo foi aprovado pelos comitês de ética locais de cada instituição.

### Variáveis do estudo

A VPA foi considerada a principal variável independente, definida pelo coeficiente de variação de três medidas da pressão arterial sistólica (PAS) obtidas durante a primeira visita do participante no período basal do ELSA-Brasil.

As variáveis sociodemográficas avaliadas foram sexo, idade, cor/raça autodeclarada (negra, parda, branca, asiática e indígena), nível educacional (ensino fundamental, ensino médio, ou superior), e renda familiar per capita, em reais.

As variáveis de risco cardiovascular avaliadas foram obesidade abdominal (circunferência da cintura > 102 cm para homens, > 88 cm para mulheres), hipertensão, diabetes, tabagismo, hipercolesterolemia (LDL ≥ 130mg/dL), hipertrigliceridemia (>150 mg/dL), taxa de filtração glomerular reduzida (<60mL/min), e velocidade de onda de pulso (m/s).

Para estimar o risco de um primeiro episódio de acidente vascular encefálico ou IAM (fatal/não fatal) ou morte por doença cardiovascular entre os participantes, durante um período de 10 anos, utilizou-se o estimador de risco de doença cardiovascular aterosclerótica (ASCVD), que foi considerado a variável dependente neste estudo. Desenvolvido pela força tarefa da *American Heart Association* em 2013, esse escore foi gerado com dados de coortes de indivíduos afro-americanos e brancos, com idade entre 40 e 79 anos, sem história prévia de doença cardiovascular, acompanhados prospectivamente por um mínimo de 12 anos. Métodos estatísticos foram implementados para a obtenção e validação das equações logarítmicas internas para estimativas específicas de risco de acordo com raça e sexo. As variáveis incluídas para estimar o risco foram idade, colesterol total, colesterol HDL, pressão sistólica, bem como um diagnóstico de diabetes e tabagismo. Indivíduos com um risco estimado em ≥7,5% são considerados de alto risco, e aqueles com risco estimado <7,5% são considerados de baixo risco para acidente vascular encefálico, IAM, ou morte cardiovascular durante os próximos 10 anos. ^33^

Informações detalhadas sobre os procedimentos laboratoriais e a metodologia usada para medir a velocidade de onda de pulso podem ser encontradas em publicações prévias. ^34 , 35^

A hipertensão foi determinada pela média aritmética das duas últimas medidas quando a PAS era ≥ 140 mmHg ou a pressão arterial diastólica era ≥ 90 mmHg, ou se o indivíduo usava medicação anti-hipertensiva. Diabetes foi definida por um diagnóstico clínico prévio, uso de medicamentos hipoglicemiantes, glicemia de jejum ≥ 126 mg/dl, hemoglobina glicada ≥ 6,5% ou glicemia pós-prandial ≥ 200 mg/dL. ^34^

O coeficiente de variação (desvio padrão/média x 100) das três medidas de PAS obtidas de cada indivíduo foi calculado no período basal e dividido em quartis. Variáveis sociodemográficas e de risco cardiovascular, bem como risco ASCVD, foram estratificados segundo cada quartil, e expressas em média e desvio padrão, ou porcentagens.

### Aferição da pressão arterial

A pressão arterial foi medida utilizando-se um oscilômetro validado, o Omron HEM 705CPINT. ^35^

Para as medidas, os pacientes encontravam-se sentados, com a bexiga vazia, e sem comer, beber, fumar ou se exercitar por pelo menos 30 minutos. O tamanho do manguito foi selecionado de acordo com a circunferência do braço. A artéria braquial foi localizada por palpação entre o tríceps e o bíceps, com o manguito colocado 2 cm acima da fossa cubital, centralizado sobre a artéria braquial. Três medidas foram obtidas em intervalos de um minuto, preferencialmente no braço esquerdo, enquanto os participantes permaneciam sentados confortavelmente sem cruzarem as pernas. Todo esforço foi feito para se obter leituras precisas e minimizar erros de medidas, e o treinamento incluiu protocolos de teste e reteste para se assegurar que condições similares fossem aplicadas a todos os participantes. O coeficiente de correlação Kappa para PAS e pressão arterial diastólica foram 0,88 (IC95%: 0,82-0,91) e 0,89 (IC95%: 0,83-0,92), respectivamente. ^7^

### Análise estatística

As variáveis categóricas foram descritas como frequências (porcentagens). O teste de Shapiro Wilk foi usado para testar a normalidade da distribuição dos dados. As variáveis contínuas foram descritas em média e desvio padrão (DP) ou mediana e intervalo interquartil (IIQ; percentis 25 ao 75), de acordo com a distribuição dos dados. As variáveis categóricas foram descritas como proporções e comparadas pelo teste do qui-quadrado de Pearson. O teste ANOVA foi usado para comparação das médias, e o teste de Kruskal-Wallis para comparação das medianas. Para estimar associações entre risco de doença cardiovascular e VPA, realizou-se análise bivariada (teste qui-quadrado de Pearson e o teste qui-quadrado de tendência). Análise multivariada foi realizada por regressão logística. As covariáveis foram testadas como potenciais modificadores de efeito e, quando isso não se confirmou no modelo, foram testadas como potenciais confundidores. As variáveis de confusão foram identificadas quando se detectou uma variância de 10% ou mais em relação aos valores de *odds ratio* (OR), correspondendo a associações entre VPA e risco cardiovascular. Para analisar as variáveis como potenciais modificadores do efeito, adotou-se o procedimento de regressão ( *backward* ) no modelo de regressão logística, o que permitiu o cálculo do OR e intervalos de confiança de 95% (IC95%). Para avaliar a modificação do efeito, o teste de razão de verossimilhança foi usado, comparando-se o modelo completo com o modelo reduzido – sem o(s) termo(s) do produto. O nível de significância adotado no estudo foi de 5%. Para a análise estatística dos dados, utilizou-se o programa STATA (Stata Corporation, College Station, Texas, EUA), versão 14.0 ^®^ .

## Resultados

Foram incluídos na análise 14.357 indivíduos, dos quais 7.884 eram mulheres e 6.473 eram homens, com idade média de 51,7 anos. Na Tabela 1 , estão descritas as características clínicas e demográficas dos indivíduos estudados, estratificados de acordo com os quartis determinados para o coeficiente de variação da pressão arterial no período basal. O sexo feminino foi predominante tanto na população total (54,1%) como em todos os quartis. Em relação à raça/cor autorrelatada, a branca foi a predominante em todos os quartis. A maioria dos participantes de cada quartil tinha nível universitário ou superior. A renda aumentou gradualmente nos quartis, com o nível mais alto observado no quarto quartil (p=0,010).

**Tabela 1:** Características clínicas e sociodemográficas dos participantes do período basal do estudo ELSA-Brasil segundo quartis de variabilidade da pressão arterial

Variáveis	Variabilidade da pressão sistólica (%)				Valor p

	1º quartil (0 – 1,78)	2º quartil (1,79 – 2,88)	3º quartil (2,89 – 4,34)	4º quartil (>4,34)	
Sexo (Masculino = 6473; Feminino=7883)					
Mulheres, n (%)	52,7	54,1	54,5	56,3	0,018 ^p^
Idade (anos), mediana (IIQ)	50 (44 - 57)	50 (44 - 57)	51 (45 -58)	53 (45 - 59)	<0,001 ^k^
Cor / raça, n (%)					
Negra/parda	46,4	44,3	43,4	41,6	
Branca	50,5	52,4	52,2	54,6	
Asiática	2,0	2,3	3,0	2,5	
Indígena	1,1	0,9	0,8	1,2	0,003 ^p^
Escolaridade, n (%)					
Baixa	9,3	8,2	9,6	10,1	
Média	30,8	30,6	30,1	30,8	
Superior	59,9	61,2	60,3	59,1	0,151 ^p^
Renda, mediana (IIQ)	1348,6 (691,5 - 2074,8)	1348,6 (726,1 - 2074,8)	1410,9 (726,1 - 2282,3)	1452,3,1 (726,1 - 2351,5)	0,010 ^k^
PAS (mm Hg), média (SD)	120,2±16,7	120,4±16,5	121,2±17,0	123,6/±17,9	<0,001 ^a^
PAD (mm Hg), média (SD)	76,3±10,5	76,3±10,6	76,4±10,6	77,1±10,8	0,001 ^a^
LDL colesterol (mg / dL), mediana (IIQ)	127 (107 - 150)	129 (108 - 151)	130 (109 - 154)	130 (109 - 154)	<0,001 ^k^
HDL colesterol (mg / dL), mediana (IIQ)	54 (46 - 65)	54 (46 - 65)	55 (47 - 65)	55 (47 - 65)	0,020 ^k^
Triglicerídeos (mg / dL), mediana (IIQ)	113 (81 - 163)	114 (80 - 165)	113 (81 - 166)	115 (83 - 167)	0,470 ^k^
Glicemia (mg / dL), mediana (IIQ)	104 (98 - 113)	105 (98 - 113)	105 (98 - 113)	105 (99 - 115)	0,001 ^k^
Hemoglobina glicada (%), mediana (IIQ)	5,3 (4,9 - 5,7)	5,5 (4,9 - 5,8)	5,3 (4,9 - 5,7)	5,3 (5,0 - 5,8)	<0,001 ^k^
Circunferência da cintura (cm), mediana (IIQ)	90,5 (82,4 - 99,4)	90,2 (81,5 - 99,7)	90,2 (81,6 - 98,6)	89,8 (81,9 - 98,2)	0,034 ^k^
TFG <60mL / min, (%)	3,6	3,9	4,0	5,2	0,004 ^p^
Diabetes mellitus, (%)	17,4	17,7	18,1	20,7	0,001 ^p^
Tabagismo, (%)	42,1	41,2	42,7	43,7	0,157 ^p^
Velocidade de onda de pulso (m /s), mediana (IIQ)	8,9 (8,0 - 10,0)	9,0 (8,1 - 10,0)	9,0 (8,1 - 10,2)	9,1 (8,1 - 10,3)	<0,001 ^k^
Alto risco cardiovascular (%)	23,3	23,7	25,7	30,5	<0,001 ^p^

* Variáveis categóricas expressas em número (%). As comparações foram testadas pelo teste χ2 de Pearson ( ^p^ ), ANOVA ( ^a^ ) teste de Kruskal-Wallis ( ^k^ ); IIQ: intervalo interquartil; DP: desvio padrão; TFG: taxa de filtração glomerular; PAS: pressão arterial sistólica; PAD: pressão arterial diastólica.

Os indivíduos do quarto quartil da VPA apresentaram valores maiores de medianas de idade (p<0,001), de LDL colesterol (p<0,001), glicemia (p=0,001) e hemoglobina glicada (p<0,001) em comparação ao primeiro quartil. As prevalências de diabetes e de taxa de filtração glomerular reduzida foram significativamente mais altas nos indivíduos do último quartil (p=0,001 e p=0,004, respectivamente). A mediana da velocidade de onda de pulso também foi significativamente maior nos indivíduos nesse quartil (p<0,001). A prevalência de alto risco para doença cardiovascular aterosclerótica foi significativamente maior no último quartil em comparação ao primeiro (p<0,001).

A Tabela 2 descreve a prevalência de alto risco cardiovascular entre os quartis de coeficiente de variação da PAS segundo o sexo, o qual foi considerado como um modificador do efeito na associação entre VPA e risco cardiovascular. Em geral, os homens apresentaram uma prevalência significativamente maior de alto risco cardiovascular que as mulheres (p<0,001). Uma vez que a VPA aumentou em ambos os sexos, a prevalência de risco elevado também foi maior, com as maiores diferenças observadas no último quartil de variabilidade ( Tabela 2 ).

**Tabela 2:** Prevalência de alto risco cardiovascular ( *≥* 7,5%) segundo quartil de variabilidade da pressão arterial e sexo

Quartis de variação da pressão sistólica	Prevalência de alto risco cardiovascular				Valor p*
	Sexo				

	Masculino		Feminino		

	n (%)	Prevalência (IC95%)	n (%)	Prevalência (IC95%)	
1º	1648 (25,9)	36,2 (33,8 – 38,5)	1896 (24,3)	12,1 (10,7 – 13,6)	<0,001
2º	1601 (25,1)	37,1 (34,7 – 39,5)	1937 (24,9)	12,7 (11,3 – 14,2)	<0,001
3º	1598 (25,0)	41,0 (38,6 – 43,5)	1951 (25,0)	13,2 (11,7 – 14,7)	<0,001
4º	1550 (24,0)	48,3 (45,8 – 50,8)	2011 (25,8)	17,1 (15,4 – 18,7)	<0,001
Valor p		<0,001		<0,001	

* teste Χ2 de tendência.

A Tabela 3 apresenta detalhes do modelo final da análise multivariada avaliando a associação entre risco elevado para doença cardiovascular aterosclerótica e VPA de acordo com o sexo. Comparações entre os quartis revelaram um risco cardiovascular mais alto para os homens classificados no penúltimo e no último quartil de coeficiente de VPA (OR=1,20; IC95%: 1,02 - 1,40; OR=1,46; IC95%: 1,25 - 1,71 respectivamente), e para as mulheres no último quartil (OR=1,27; IC95%: 1,03 - 1,57), após ajuste quanto aos fatores de confusão (obesidade abdominal, renda e nível educacional), incluindo média de PAS.

**Tabela 3:** Modelo final da associação entre alto risco cardiovascular (≥ 7,5%) e variabilidade da pressão arterial entre homens e mulheres

Quartis de variação da pressão sistólica	Sexo			
	Masculino		Feminino	

	OR não ajustado (IC95%)	*OR ajustado (IC95%)	OR não ajustado (IC95%)	*OR ajustado (IC95%)
1º	1,0	1,0	1,0	1,0
2º	1,04 (0,90 – 1,20)	1,03 (0,89 – 1,21)	1,06 (0,87 – 1,28)	1,08 (0,86 – 1,35)
3º	1,23 (1,06 – 1,41)	1,20 (1,02 – 1,40)	1,10 (0,91 – 1,33)	1,09 (0,87 -1,36)
4º	1,65 (1,43 – 1,90)	1,46 (1,25 – 1,71)	1,49 (1,24 – 1,78)	1,27 (1,03 – 1,57)

* Ajustado para média de pressão arterial sistólica, obesidade abdominal, renda e escolaridade

## Discussão

Nos dados coletados dos indivíduos incluídos na fase basal do estudo ELSA-Brasil, a VPA em uma mesma visita associou-se a um risco elevado de se desenvolver doença cardiovascular aterosclerótica, e a marcadores de risco cardiovascular, tais como hipercolesterolemia, diabetes, taxa de filtração glomerular reduzida e velocidade de onda de pulso elevada. A prevalência de alto risco cardiovascular aumentou progressivamente com a VPA, e foi significativamente maior em homens que em mulheres em todos os quartis avaliados. Independentemente da média da PAS, uma variação mais alta no coeficiente de VPA foi significativamente associado com risco cardiovascular para homens nos dois quartis mais altos, e para mulheres no último quartil.

O valor prognóstico da VPA em longo prazo, tanto medida por MAPA como por medidas casuais da pressão arterial, foi comprovado em estudos prévios. ^9 , 11 , 16 , 18^ Um estudo ^36^ do tipo coorte coreano, recentemente publicado, demonstrou uma associação da variabilidade de PAS, glicemia, colesterol total e índice de massa corporal com mortalidade e eventos cardiovasculares. ^36^

O valor prognóstico da VPA em curto prazo (24 horas), medida por MAPA, foi bem demonstrado quanto a lesões em órgãos-alvo e desfechos cardiovasculares em estudos transversais e longitudinais. ^11 , 14 , 15 , 23 , 24^ Contudo, há menos evidências em relação à VPA em uma mesma consulta, ^12 , 17 , 28^ destacando a necessidade de confirmação em termos das implicações clínicas. Em comparação a outros estudos que avaliaram essa questão, nossos resultados corroboraram os achados de Grassi et al., ^12^ que descreveram a relação entre a VPA em uma mesma visita e fatores de risco cardiovascular, tais como idade avançada, hipercolesterolemia, e a presença de diabetes, os quais foram significativamente mais prevalentes no último quartil do coeficiente da variação da PAS. Em um estudo ^13^ envolvendo uma população menor na Turquia, os autores avaliaram a VPA por MAPA e o coeficiente de variação da PAS, e observaram uma associação independente entre risco e VPA, porém sem diferença entre os sexos.

Além disso, encontramos uma maior frequência de taxa de filtração glomerular reduzida e de velocidade de onda de pulso mais elevada entre indivíduos com VPA mais alta. Apesar do fato de que medidas casuais da pressão arterial tenham sido usadas para avaliar VPA em curto prazo, nós identificamos uma relação com a taxa de filtração glomerular reduzida, um marcador precoce de risco para doença renal crônica, o que foi similar aos resultados de outro estudo conduzido em uma população coreana. ^28^ Um dado interessante em nosso estudo foi a observação de uma associação entre VPA na mesma visita e a velocidade de onda de pulso elevada, um marcador importante de rigidez de grandes artérias, o que corrobora resultados somente vistos em estudos em que se empregou MAPA. ^37 , 38^

A avaliação da VPA pode ser influenciada pela escolha do método e o intervalo de tempo considerado entre as medidas. ^12^ Uma revisão da literatura destacou possíveis mecanismos fisiopatológicos envolvidos na associação observada entre VPA em uma única visita e alto risco cardiovascular, incluindo aumento da atividade simpática central e da viscosidade sanguínea, redução do reflexo arterial e da complacência arterial, e mudança dos níveis séricos de angiotensina II, bradicinina, endotelina e óxido nítrico, além de fatores emocionais e comportamentais. ^26^

Os achados no presente estudo sugerem que a VPA em uma única visita pode ser considerada um importante marcador de risco cardiovascular, e que sua avaliação pode auxiliar médicos a identificarem pacientes que necessitem de um monitoramento mais próximo, ou mesmo de um tratamento mais intensivo. É importante notar que a população estudada consistiu principalmente de indivíduos normotensos (64,2%), o que reforça a ideia de que a pressão arterial seja uma variável de risco contínuo, e que a avaliação da VPA seja importante não somente entre os pacientes hipertensos. ^10 , 17^ Nossos resultados reforçam ainda a importância clínica de se monitorar a VPA, além de se obter medidas isoladas em uma única visita, dada a possibilidade de se identificar indivíduos com alto risco cardiovascular. ^28^

Nossos resultados ganham força pelo tamanho da população avaliada, pelo uso de um método simples, de baixo custo, reprodutível e eficiente para avaliar a VPA, e em determinar a associação entre VPA e o risco cardiovascular. Quanto às limitações, destacamos a utilização de uma amostra de conveniência sem randomização e a natureza transversal deste estudo que não nos permitiu determinar se o risco cardiovascular leva ao desenvolvimento de VPA, ou vice-versa.

Embora não seja possível generalizarmos nossos resultados a toda a população, é notável que nossa amostra é altamente representativa de populações urbanas de grandes capitais brasileiras, com características sociodemográficas similares às encontradas em outros centros importantes do país. De uma perspectiva futura, nós destacamos a possibilidade de se avaliar a associação entre variabilidade da PAS em uma única visita e eventos cardiovasculares fatais e não fatais entre os participantes do ELSA-Brasil. Os autores ainda sugerem que mais estudos são necessários, como ensaios clínicos randomizados utilizando diferentes classes de drogas anti-hipertensivas, na tentativa de se determinar o impacto desses tratamentos sobre a VPA, bem como estabelecer associações com desfechos cardiovasculares e mortalidade.

## Conclusão

A maior variabilidade da PAS em uma mesma visita nos participantes do período basal do ELSA-Brasil foi associada a um risco cardiovascular mais alto, principalmente entre homens, independentemente da média da PAS.

## References

[1] NCD Risk Factor Collaboration (NCD-RisC) (2017). Worldwide Trends in Blood Pressure from 1975 to 2015: A Pooled Analysis of 1479 Population-Based Measurement Studies with 19•1 Million Participants. Lancet.

[2] GBD 2017 Causes of Death Collaborators (2018). Global, Regional, and National Age-Sex-Specific Mortality for 282 Causes of Death in 195 Countries and Territories, 1980-2017: A Systematic Analysis for the Global Burden of Disease Study 2017. Lancet.

[3] Lewington S, Clarke R, Qizilbash N, Peto R, Collins R, Prospective Studies Collaboration (2002). Age-specific Relevance of Usual Blood Pressure to Vascular Mortality: A Meta-Analysis of Individual Data for One Million Adults in 61 Prospective Studies. Lancet.

[4] Magalhães LB, Amorim AM, Rezende EP (2018). Conceito e aspectos epidemiológicos da hipertensão arterial. Rev Bras Hipertens.

[5] Andrade SSA, Stopa SR, Brito AS, Chueri PS, Szwarcwald CL, Malta DC (2015). Prevalência de hipertensão arterial autorreferida na população brasileira: análise da Pesquisa Nacional de Saúde, 2013. Epidemiol Serv Saúde.

[6] Picon RV, Fuchs FD, Moreira LB, Riegel G, Fuchs SC (2012). Trends in Prevalence of Hypertension in Brazil: A Systematic Review with Meta-Analysis. PLoS One.

[7] Chor D, Ribeiro ALP, Carvalho MS, Duncan BB, Lotufo PA, Nobre AA (2015). Prevalence, Awareness, Treatment and Influence of Socioeconomic Variables on Control of High Blood Pressure: Results of the ELSA-Brasil Study. PLoS One.

[8] Rothwell PM (2010). Limitations of the Usual Blood-Pressure Hypothesis and Importance of Variability, Instability, and Episodic Hypertension. Lancet.

[9] Rothwell PM, Howard SC, Dolan E, O’Brien E, Dobson JE, Dahlöf B (2010). Prognostic Significance of Visit-to-Visit Variability, Maximum Systolic Blood Pressure, and Episodic Hypertension. Lancet.

[10] Mancia G, Bombelli M, Facchetti R, Madotto F, Corrao G, Trevano FQ (2007). Long-Term Prognostic Value of Blood Pressure Variability in the General Population: Results of the Pressioni Arteriose Monitorate e Loro Associazioni Study. Hypertension.

[11] Wan EY, Fung CS, Yu EY, Fong DY, Chen JY, Lam CL (2017). Association of Visit-to-Visit Variability of Systolic Blood Pressure With Cardiovascular Disease and Mortality in Primary Care Chinese Patients With Type 2 Diabetes-A Retrospective Population-Based Cohort Study. Diabetes Care.

[12] Grassi G, Seravalle G, Maloberti A, Facchetti R, Cuspidi C, Bombelli M (2015). Within-Visit BP Variability, Cardiovascular Risk Factors, and BP Control in Central and Eastern Europe: Findings from the BP-CARE study. J Hypertens.

[13] Celik M, Yuksel UC, Yildirim E, Gursoy E, Koklu M, Yasar S (2016). The Relationship Between Blood Pressure Variability and Pooled Cohort Risk Assessment Equations 10-year Cardiovascular Risk Score. Blood Press Monit.

[14] Sega R, Corrao G, Bombelli M, Beltrame L, Facchetti R, Grassi G (2002). Blood Pressure Variability and Organ Damage in a General Population: Results from the PAMELA Study (Pressioni Arteriose Monitorate E Loro Associazioni). Hypertension.

[15] Leoncini G, Viazzi F, Storace G, Deferrari G, Pontremoli R (2013). Blood Pressure Variability and Multiple Organ Damage in Primary Hypertension. J Hum Hypertens.

[16] Mancia G, Facchetti R, Parati G, Zanchetti A (2012). Visit-to-Visit Blood Pressure Variability, Carotid Atherosclerosis, and Cardiovascular Events in the European Lacidipine Study on Atherosclerosis. Circulation.

[17] Ribeiro AH, Lotufo PA, Fujita A, Goulart AC, Chor D, Mill JG (2017). Association Between Short-Term Systolic Blood Pressure Variability and Carotid Intima-Media Thickness in ELSA-Brasil Baseline. Am J Hypertens.

[18] Yano Y, Fujimoto S, Kramer H, Sato Y, Konta T, Iseki K (2015). Long-Term Blood Pressure Variability, New-Onset Diabetes Mellitus, and New-Onset Chronic Kidney Disease in the Japanese General Population. Hypertension.

[19] Sarafidis PA, Ruilope LM, Loutradis C, Gorostidi M, de la Sierra A, de la Cruz JJ (2018). Blood Pressure Variability Increases with Advancing Chronic Kidney Disease Stage: A Cross-Sectional Analysis of 16 546 Hypertensive Patients. J Hypertens.

[20] Stevens SL, Wood S, Koshiaris C, Law K, Glasziou P, Stevens RJ (2016). Blood Pressure Variability and Cardiovascular Disease: Systematic Review and Meta-Analysis. BMJ.

[21] Juhanoja EP, Niiranen TJ, Johansson JK, Puukka PJ, Thijs L, Asayama K (2017). Outcome-Driven Thresholds for Increased Home Blood Pressure Variability. Hypertension.

[22] Kostis JB, Sedjro JE, Cabrera J, Cosgrove NM, Pantazopoulos JS, Kostis WJ (2014). Visit-to-Visit Blood Pressure Variability and Cardiovascular Death in the Systolic Hypertension in the Elderly Program. J Clin Hypertens.

[23] Manios E, Tsagalis G, Tsivgoulis G, Barlas G, Koroboki E, Michas F (2009). Time Rate of Blood Pressure Variation is Associated with Impaired Renal Function in Hypertensive Patients. J Hypertens.

[24] Morano A, Ravera A, Agosta L, Sappa M, Falcone Y, Fonte G (2018). Extent of, and Variables Associated with, Blood Pressure Variability Among Older Subjects. Aging Clin Exp Res.

[25] Irigoyen MC, Angelis K, Santos F, Dartora DR, Rodrigues B, Consolim-Colombo FM (2016). Hypertension, Blood Pressure Variability, and Target Organ Lesion. Curr Hypertens Rep.

[26] Parati G, Ochoa JE, Lombardi C, Bilo G (2013). Assessment and Management of Blood-Pressure Variability. Nat Rev Cardiol.

[27] Rothwell PM, Howard SC, Dolan E, O’Brien E, Dobson JE, Dahlöf B (2010). Effects of Beta Blockers and Calcium-Channel Blockers on Within-Individual Variability in Blood Pressure and Risk of Stroke. Lancet Neurol.

[28] Shin JH, Shin J, Kim BK, Lim YH, Park HC, Choi SI (2013). Within-Visit Blood Pressure Variability: Relevant Factors in the General Population. J Hum Hypertens.

[29] Muntner P, Levitan EB, Reynolds K, Mann DM, Tonelli M, Oparil S (2012). Within-Visit Variability of Blood Pressure and All-Cause and Cardiovascular Mortality Among US Adults. J Clin Hypertens.

[30] Schutte R, Thijs L, Liu YP, Asayama K, Jin Y, Odili A (2012). Within-Subject Blood Pressure Level--Not Variability--Predicts Fatal and Nonfatal Outcomes in a General Population. Hypertension.

[31] Aquino EM, Barreto SM, Bensenor IM, Carvalho MS, Chor D, Duncan BB (2012). Brazilian Longitudinal Study of Adult Health (ELSA-Brasil): Objectives and Design. Am J Epidemiol.

[32] Aquino EM, Araujo MJ, Almeida Mda C, Conceição P, Andrade CR, Cade NV (2013). Participants Recruitment in ELSA-Brasil (Brazilian Longitudinal Study for Adult Health). Rev Saude Publica.

[33] Committee ACC Guidelines (2014). Reply: 2013 ACC/AHA Guideline on the Assessment of Cardiovascular Risk. J Am Coll Cardiol.

[34] Schmidt MI, Duncan BB, Mill JG, Lotufo PA, Chor D, Barreto SM (2015). Cohort Profile: Longitudinal Study of Adult Health (ELSA-Brasil). Int J Epidemiol.

[35] Mill JG, Pinto K, Griep RH, Goulart A, Foppa M, Lotufo PA (2013). Medical Assessments and Measurements in ELSA-Brasil. Rev Saude Publica.

[36] Kim MK, Han K, Park YM, Kwon HS, Kang G, Yoon KH (2018). Associations of Variability in Blood Pressure, Glucose and Cholesterol Concentrations, and Body Mass Index With Mortality and Cardiovascular Outcomes in the General Population. Circulation.

[37] Tedla YG, Yano Y, Carnethon M, Greenland P (2017). Association Between Long-Term Blood Pressure Variability and 10-Year Progression in Arterial Stiffness: The Multiethnic Study of Atherosclerosis. Hypertension.

[38] Boardman H, Lewandowski AJ, Lazdam M, Kenworthy Y, Whitworth P, Zwager CL (2017). Aortic Stiffness and Blood Pressure Variability in Young People: A Multimodality Investigation of Central and Peripheral Vasculature. J Hypertens.

