# Supplementary material for: Variabilidade da Pressão Arterial em Única Visita e Risco Cardiovascular em Participantes do ELSA-Brasil
Source: Arq Bras Cardiol. 2022 Aug 24;119(4):505–11. [Article in Portuguese] doi: 10.36660/abc.20210804 (PMC9563895; doi:10.36660/abc.20210804)
Supplement: Translation

# Translation: Within-Visit Blood Pressure Variability and Cardiovascular Risk in ELSA-Brasil Study Participants

**Authors:** André Sant’Anna Zarife, Helena Fraga-Maia, José Geraldo Mill, Paulo Lotufo, Rosane Harter Griep, Maria de Jesus Mendes da Fonseca, Luciara Leite Brito, Maria da Conceição Almeida, Roque Aras, Sheila Maria Alvim Matos

**Affiliations:** 1 Brazil Universidade Federal da Bahia – UFBA, Salvador, BA – Brazil; 2 Brazil Hospital Geral Roberto Santos, Salvador, BA – Brazil; 3 Brazil Universidade do Estado da Bahia – UNEB, Salvador, BA – Brazil; 4 Brazil Universidade Federal do Espírito Santo – Centro de Ciências da Saúde, Vitoria, ES – Brazil; 5 Brazil Universidade de São Paulo, São Paulo, SP – Brazil; 6 Brazil Fundação Oswaldo Cruz, Rio de Janeiro, RJ – Brazil; 7 Brazil Centro de Pesquisas Gonçalo Moniz, Salvador, BA – Brazil

**Keywords:** Arterial Pressure, Heart Disease Risk Factors, Hypertension

## Abstract

**Background:**

Blood pressure variability (BPV) is of prognostic value for fatal and non-fatal cardiovascular outcomes.

**Objective:**

This study aimed to evaluate the association between within-visit BPV and cardiovascular risk among participants of the Brazilian Longitudinal Study of Adult Health (ELSA-Brasil).

**Methods:**

The present cross-sectional study was carried out using baseline data (2008-2010) of 14,357 ELSA-Brasil participants with no prior history of cardiovascular disease. Within-visit BPV was quantified by the coefficient of variation of three standardized systolic blood pressure (SBP) measurements using an oscillometer. Anthropometric measurements and laboratory tests were also performed. Cardiovascular risk was assessed using the atherosclerotic cardiovascular disease risk estimator (ASCVD) and multivariate logistic regression analysis was employed with a significance level of 5%.

**Results:**

Significantly higher cardiovascular risk was determined by increased BPV for both sexes. A significantly higher prevalence of high risk was found in men than women across all quartiles, with the highest difference observed in the fourth quartile of variability (48.3% vs. 17.1%). Comparisons among quartiles in each sex revealed a significantly higher cardiovascular risk for men in the third (OR=1.20; 95%CI: 1.02 - 1.40) and fourth quartiles (OR=1.46; 95%CI: 1.25 -1.71), and for women in the fourth quartile (OR=1.27; 95%CI: 1.03 - 1.57).

**Conclusion:**

Analysis of baseline data of the ELSA-Brasil participants revealed that blood pressure variability was associated with increased cardiovascular risk, especially in men.

## Introduction

Hypertension is considered the main risk factor for cardiovascular disease, with increasing global mortality evidenced in recent years.^1 - 3^ In Brazil, it is estimated that arterial hypertension affects approximately 36 million Brazilians, with epidemiological studies reporting a prevalence ranging from 21.4 - 35.8%.^4 - 7^

Although arterial hypertension is considered an important risk factor for stroke and acute myocardial infarction (AMI), evidence suggests that elevated blood pressure is not the only relevant pathophysiological factor involved in cardiovascular events.^8 , 9^ Several studies have demonstrated the importance of blood pressure variability (BPV) in the association between arterial hypertension and cardiovascular risk.^10 - 24^ BPV is a complex phenomenon, in which fluctuations in blood pressure readings can be influenced by the individual’s environment, behavior, hormonal and central nervous system activity, among other factors.

BPV is assessed beat-by-beat through intra-arterial measurements, by physicians in a clinical setting, by using an ambulatory blood pressure monitoring (ABPM) device, or a home blood pressure monitor (HBPM) at very short, short, medium or long intervals.^25 , 26^ Short-term (24 hours), medium-term (2+ days) and long term (at weekly, monthly or yearly intervals) BPV is associated with high cardiovascular risk,^12 , 13^ left ventricular hypertrophy,^14 , 15^ increased carotid intima-media thickness,^16 , 17^ chronic renal failure,^18 , 19^ and fatal and non-fatal cardiovascular events.^20 - 22^

Some studies have demonstrated that the within-visit BPV can be positively associated with stroke and cardiovascular risk,^27 , 28^ while others found no associations with cardiovascular or total mortality outcomes.^29 , 30^ Previously published data from the Brazilian Longitudinal Study on Adult Health (ELSA-Brasil) reported an association between within-visit BPV and carotid intima-media thickness.^17^ Despite the body of accumulated knowledge in the literature, it remains impossible to conclude that BPV represents an independent risk factor that should be modulated and controlled through antihypertensive treatment, or whether it is simply a marker that accompanies elevated blood pressure.^26^ Thus, the present study aimed to establish associations between BPV in a single visit and cardiovascular risk in a cohort of participants at baseline of the ELSA-Brasil.^31^

## Methods

### Study design

The present cross-sectional study was carried out with baseline data (2008-2010) from the ELSA-Brasil. ELSA-Brasil is a cohort study initiated in 2008 involving public servants at higher education and research institutions located in six Brazilian capitals (Salvador, Vitória, Belo Horizonte, Porto Alegre, São Paulo, and Rio de Janeiro) and aims to investigate the incidence and progression of cardiovascular diseases and diabetes, as well as associated biological, environmental, psychological, and social factors. At baseline, data collection consisted of interviews, anthropometric measurements, clinical examinations and the collection of biological samples. Participants are contacted by phone annually to record health events, and every four years they are called back for new interviews and assessment of health status and outcomes.^32^

### Population

Active and retired public servants from six institutions were included, aged between 35 and 74 years. Individuals with severe cognitive or communication impairment were considered ineligible, as well as those who intended to retire in the near future or had retired and then moved to a residence far from their respective local research center. Also, women who were pregnant or had given birth less than four months prior to their baseline visit were ineligible to participate. Of the 15,105 ELSA-Brasil baseline participants, we excluded 749 (5.0%) who self-reported previous stroke, myocardial infarction, revascularization, or heart failure. As a result, our study sample comprised 14,357 individuals. All participants signed an informed consent form, and the study protocol was approved by the institutional review board of each institution.

### Study variables

BPV was considered the main independent variable, defined by the coefficient of variation of three systolic blood pressure (SBP) measurements obtained during the first visit of each participant at baseline.

The sociodemographic variables evaluated were sex, age, self-declared race/skin color (black, brown, white, asian, indigenous), education level (elementary school, high school or university degree) and per capita family income in Brazilian reals.

The cardiovascular risk variables evaluated were abdominal obesity (waist circumference >102 cm for men, >88 cm for women), hypertension, diabetes, smoking, hypercholesterolemia (LDL ≥130 mg/dL), hypertriglyceridemia (>150 mg/dL), reduced glomerular filtration rate (<60 mL/min), and pulse wave velocity (m/s).

To estimate the risk of a first episode of stroke or AMI (fatal / non-fatal) or cardiovascular death among the study participants over a 10-year period, the risk estimator of cardiovascular atherosclerotic disease (ASCVD) was used, which was considered the dependent variable in this study. Developed by an American Heart Association task force in 2013, this score was generated using data obtained from cohorts that included African-American and white individuals, aged 40-79 years, with no previous history of cardiovascular disease, and who were prospectively followed for a minimum of 12 years.

Statistical methods were implemented to obtain and validate the internal logarithmic equations for specific risk estimates according to sex and race. The variables included to estimate the risk were age, total cholesterol, HDL cholesterol and systolic pressure, as well as a diagnosis of diabetes and smoking habit. Individuals with a risk estimated at ≥7.5% are considered to be at high risk, while those <7.5% are considered to be at low risk for stroke, AMI or cardiovascular death over the next 10 years.^33^

Detailed information on laboratory testing procedures and the methodology used to measure pulse wave velocity can be found in previous publications.^34 , 35^

Hypertension was determined by the arithmetic mean of the last two measurements when SBP was ≥ 140 mmHg or diastolic blood pressure ≥ 90 mmHg, or if the subject used antihypertensive medication. Diabetes was defined by a previous clinical diagnosis, use of antidiabetic medications, fasting glucose ≥126 mg/dl, glycated hemoglobin ≥ 6.5% or postprandial glucose ≥ 200 mg /dL.^34^

The coefficient of variation (standard deviation/mean x 100) of the three SBP measurements obtained for each individual was calculated at baseline of the study and divided into quartiles. Sociodemographic and cardiovascular risk variables, as well as ASCVD risk, were stratified according to each quartile, and expressed as means and standard deviation, or percentages.

### Blood pressure measurement

A validated oscillometer device, Omron HEM 705CPINT, was used to measure arterial blood pressure.^35^

Measurements were performed in sitting position, with an empty bladder, and without eating, drinking, smoking or exercising for at least 30 minutes before the measurement. Cuff size was selected according to arm circumference. The brachial artery was detected by palpation between the triceps and biceps, with the cuff placed 2 cm above the cubital fossae, centered over the brachial artery. Three measurements were obtained at one-minute intervals, preferably on the left arm, while the participant was seated comfortably without crossing their legs. Every effort was made to obtain accurate readings and minimize measurement errors, and training included test-retest protocols to ensure that similar conditions would be used for all participants. The Kappa correlation coefficient for SBP and diastolic blood pressure were 0.88 (95%CI: 0.82-0.91) and 0.89 (95%CI: 0.83-0.92), respectively.^7^

### Statistical analysis

Categorical variables were described as frequencies (percentages). The Shapiro-Wilk test was used to test normality of data distribution. Continuous variables were described as mean and standard deviation (SD) or median and interquartile range (IQR; 25th to 75th percentile), according to data distribution. Categorical variables were described by proportions and compared using Pearson’s chi-squared test. The ANOVA test was used to compare the means, and the Kruskal-Wallis test for the medians. To estimate associations between risk of cardiovascular disease and BPV, bivariate analysis was performed (Pearson’s chi-squared test and chi-square test for trend). Lastly, multivariate analysis was performed by logistic regression. Covariates were tested as potential effect modifiers; when not confirmed in the model as such, they were tested as potential confounders. Confounding variables were identified when a variance of 10% or more was detected with respect to odds ratio (OR) values corresponding to associations between BPV and cardiovascular risk. To analyze the variables as potential effect modifiers, the backward procedure was adopted in the logistic regression model, which allowed the estimation of OR and respective 95% confidence intervals (95% CI). To assess the effect modification, the likelihood-ratio test was used, comparing the complete model with the reduced model - without the product term (s). The level of significance admitted in the study was 5%. For the statistical analysis of the data, the STATA (Stata Corporation, College Station, Texas, USA), version 14.0^®^software was used.

## Results

A total of 14,357 individuals were included in the analysis, 7,884 of whom were females and 6,473 were males, with a mean age of 51.7 years. Table 1 lists the sociodemographic and clinical characteristics of the studied individuals, stratified according to the quartiles determined for the BP coefficient of variation at baseline. Females predominated in both the overall population (54.1%), as well as in all quartiles. With respect to self-reported race/skin color, white was the most predominant across all quartiles. Most participants had university degree or higher in each quartile. Income level gradually increased across the quartiles, with the highest level seen in the fourth quartile (p=0.010).

**Table 1:** Sociodemographic and clinical characteristics of participants in the ELSA-Brasil baseline according to blood pressure variability quartiles

Variables	Systolic blood pressure variability (%)				p-value

	1^st^quartile (0 – 1.78)	2^nd^quartile (1.79 – 2.88)	3^rd^quartile (2.89 – 4.34)	4^th^quartile (>4.34)	
Sex (Male=6,473; Female=7,883)					
Female, n (%)	52.7	54.1	54.5	56.3	0.018^p^
Age (years), median (IQR)	50 (44 - 57)	50 (44 - 57)	51 (45 -58)	53 (45 - 59)	<0.001^k^
Skin color, n (%)					
Black/brown	46.4	44.3	43.4	41.6	
White	50.5	52.4	52.2	54.6	
Asian	2.0	2.3	3.0	2.5	
Indigenous	1.1	0.9	0.8	1.2	0.003^p^
Schooling, n (%)					
Low	9.3	8.2	9.6	10.1	
Medium	30.8	30.6	30.1	30.8	
Superior	59.9	61.2	60.3	59.1	0.151^p^
Income, median (IQR)	1348.6 (691.5 - 2074.8)	1348.6 (726.1 - 2074.8)	1410.9 (726.1 - 2282.3)	1452.3.1 (726.1 - 2351.5)	0.010^k^
SBP (mm Hg), mean (SD)	120.2±16.7	120.4±16.5	121.2±17.0	123.6/±17.9	<0.001^a^
DBP (mm Hg), mean (SD)	76.3±10.5	76.3±10.6	76.4±10.6	77.1±10.8	0.001^a^
LDL cholesterol (mg / dL), median (IQR)	127 (107 - 150)	129 (108 - 151)	130 (109 - 154)	130 (109 - 154)	<0.001^k^
HDL cholesterol (mg / dL), median (IQR)	54 (46 - 65)	54 (46 - 65)	55 (47 - 65)	55 (47 - 65)	0.020^k^
Triglycerides (mg / dL), median (IQR)	113 (81 - 163)	114 (80 - 165)	113 (81 - 166)	115 (83 - 167)	0.470^k^
Blood glucose (mg / dL), median (IQR)	104 (98 - 113)	105 (98 - 113)	105 (98 - 113)	105 (99 - 115)	0.001^k^
Glycated hemoglobin (%), median (IQR)	5.3 (4.9 - 5.7)	5.5 (4.9 - 5.8)	5.3 (4.9 - 5.7)	5.3 (5.0 - 5.8)	<0.001^k^
Waist circumference (cm), median (IQR)	90.5 (82.4 - 99.4)	90.2 (81.5 - 99.7)	90.2 (81.6 - 98.6)	89.8 (81.9 - 98.2)	0.034^k^
GFR <60ml / min, (%)	3.6	3.9	4.0	5.2	0.004^p^
Diabetes mellitus, (%)	17.4	17.7	18.1	20.7	0.001^p^
Smoking, (%)	42.1	41.2	42.7	43.7	0.157^p^
Pulse wave velocity (m / s), median (IQR)	8.9 (8.0 - 10.0)	9.0 (8.1 - 10.0)	9.0 (8.1 - 10.2)	9.1 (8.1 - 10.3)	<0.001^k^
High cardiovascular risk (%)	23.3	23.7	25.7	30.5	<0.001^p^

^*^Categorical variables expressed as number (%). Comparisons were made by Pearson’s χ2 (^p^), ANOVA (^a^) or Kruskal-Wallis test (^k^); IQR: interquartile range; SD: standard deviation; GFR: glomerular filtration rate; SBP: systolic blood pressure; DBP: diastolic blood pressure.

Individuals of the fourth quartile of BPV presented significantly higher median age (p<0.001), higher median LDL cholesterol levels (p<0.001), blood glucose (p=0.001) and glycated hemoglobin (p<0.001) in comparison to the first quartile. The prevalence of diabetes and reduced glomerular filtration rate were significantly higher among individuals in the last quartile (p=0.001 and p=0.004, respectively). The median pulse wave velocity was also significantly higher among individuals in this quartile (p<0.001). The prevalence of high risk of atherosclerotic cardiovascular disease was significantly higher in the last quartile compared to the first (p<0.001).

Table 2 describes the prevalence of an elevated risk of developing atherosclerotic cardiovascular disease among the quartiles of SBP coefficient of variation according to sex, which was considered as a modifier of effect in the association between BPV and cardiovascular risk. In general, men presented a significantly higher prevalence of high ASCVD risk than women (p <0.001). As BPV increased in both sexes, the prevalence of high risk was also higher, with the largest differences seen in the last quartile of variability ( Table 2 ).

**Table 2:** Prevalence of high cardiovascular risk (≥ 7.5%) according to quartile of blood pressure variability and sex

Systolic blood pressure variation quartiles	Prevalence of high cardiovascular risk				p-value*
	Sex				

	Male		Female		

	n (%)	Prevalence (95CI%)	n (%)	Prevalence (95%CI)	
1^st^	1648 (25.9)	36.2 (33.8 – 38.5)	1896 (24.3)	12.1 (10.7 – 13.6)	<0.001
2^nd^	1601 (25.1)	37.1 (34.7 – 39.5)	1937 (24.9)	12.7 (11.3 – 14.2)	<0.001
3^rd^	1598 (25.0)	41.0 (38.6 – 43.5)	1951 (25.0)	13.2 (11.7 – 14.7)	<0.001
4^th^	1550 (24.0)	48.3 (45.8 – 50.8)	2011 (25.8)	17.1 (15.4 – 18.7)	<0.001
p value		<0.001		<0.001	

* Χ2 trend test

Table 3 details the final model of the multivariate analysis assessing the association between high atherosclerotic cardiovascular risk and BPV according to sex. Comparisons among the quartiles revealed a significantly higher overall cardiovascular risk for men classified in the penultimate and last BPV coefficient quartiles (OR=1.20; 95%CI: 1.02 - 1.40; OR=1.46; 95%CI: 1.25 -1.71, respectively), and for women in the last quartile (OR=1.27; 95%CI: 1.03 - 1.57), after adjusting for confounders (abdominal obesity, income and education level), including mean SBP.

**Table 3:** Final model of the association between high cardiovascular risk and blood pressure variability between men and women

Systolic blood pressure variation quartiles	Sex			
	Male		Female	

	Crude OR (95CI%)	*Adjusted OR (95CI%)	Crude OR (95CI%)	*Adjusted OR (95CI%)
1^st^	1.0	1.0	1.0	1.0
2^nd^	1.04 (0.90 – 1.20)	1.03 (0.89 – 1.21)	1.06 (0.87 – 1.28)	1.08 (0.86 – 1.35)
3^rd^	1.23 (1.06 – 1.41)	1.20 (1.02 – 1.40)	1.10 (0.91 – 1.33)	1.09 (0.87 -1.36)
4^th^	1.65 (1.43 – 1.90)	1.46 (1.25 – 1.71)	1.49 (1.24 – 1.78)	1.27 (1.03 – 1.57)

* Adjusted for average systolic pressure, abdominal obesity, income and education

## Discussion

In the data collected from individuals included the ELSA-Brasil, within-visit BPV was found to be associated with a high risk of developing atherosclerotic cardiovascular disease, and was related to markers of cardiovascular risk, such as hypercholesterolemia, diabetes, reduced glomerular filtration rate and high pulse wave velocity. The prevalence of high cardiovascular risk progressively increased with BPV, and was observed to be significantly higher among men compared to women in all quartiles evaluated. Regardless of mean SBP, a higher blood pressure coefficient of variation was significantly associated with cardiovascular risk for men in the two highest quartiles, and in the last quartile for women.

The prognostic value of long-term BPV, both through ABPM and casual blood pressure measurements, has been proven in previous studies.^9 , 11 , 16 , 18^ A recently published Korean cohort study^36^ demonstrated an association of SBP, blood glucose, total cholesterol and body mass index variability with mortality and cardiovascular events.^36^

The prognostic value of short-term (24-hour) BPV, as measured by ABPM, has been extensively demonstrated regarding target organ damage and cardiovascular outcomes in cross-sectional and longitudinal studies.^11 , 14 , 15 , 23 , 14^ However, there is less evidence regarding BPV in a single consultation,^12 , 17 , 28^ highlighting the need for further confirmation in terms of clinical implications. Compared to other studies that evaluated this issue, our results corroborated findings reported by Grassi et al.,^12^ who described the relationship between within-visit BPV and cardiovascular risk factors, such as advanced age, hypercholesterolemia and the presence of diabetes, which were significantly more prevalent in the last quartile of the SBP coefficient of variation. In contrast to the results reported by Grassi et al., we demonstrated a positive relationship between elevated cardiovascular risk and BPV among both sexes, which was shown to be stronger in men. Another study^13^ involving a smaller-sized population in Turkey evaluated BPV by ABPM and SBP coefficient of variation, observing an independent association between risk and BPV, with no differences between sexes though.

We additionally found a higher prevalence of reduced glomerular filtration rate and higher pulse wave velocity among individuals with higher BPV. Despite the fact that casual blood pressure measurements were used to assess short-term BPV, we did identify a link with reduced glomerular filtration rate, an early risk marker of chronic kidney disease, which was similar to results from another study conducted in a Korean population.^28^ Interestingly, the association observed herein between within-visit BPV and elevated pulse wave velocity, an important marker of stiffness in large arteries, corroborated results only seen in studies employing ABPM.^37 , 38^

The assessment of BPV can be influenced by the choice of method and the time interval considered between measurements.^12^ A review of the literature highlighted possible pathophysiological mechanisms involved in the association observed between short-term BPV in a single visit and high cardiovascular risk, including increased central sympathetic activity, decreased arterial and cardiopulmonary reflexes, increased blood viscosity, decreased arterial compliance, changes in serum insulin levels, angiotensin II, bradykinins, endothelin and nitric oxide, in addition to emotional and behavioral factors.^26^

The findings here suggest that BPV in a single visit can be considered an important marker of cardiovascular risk, and that the evaluation of this parameter can help physicians to identify patients who should be monitored more closely, as well as those that may even require more intensive treatment. It is important to note that the population studied consisted mainly of normotensive individuals (64.2%), which reinforces the notion that blood pressure is a continuous risk variable and that the assessment of BPV is important not only among hypertensive patients.^10 , 17^ Our results further reinforce the clinical importance of monitoring BPV, in addition to obtaining isolated measures in a single visit, given the possibility of identifying individuals with high cardiovascular risk.^28^

Our results are strengthened by the size of the population evaluated, the use of a simple, low-cost, reproducible and efficient method to assess BPV, and in determining the association between BPV and cardiovascular risk. With regard to limitations, a convenience sample was employed without randomization, and the cross-sectional nature of this study prevents us from determining whether cardiovascular risk lead to development BPV, or vice-versa. Although it is not feasible to generalize our results to the overall population, it is notable that our sample is highly representative of urban populations from large Brazilian capitals, with similar sociodemographic characteristics as those found in other major centers throughout the country. From a future perspective, we highlight the possibility of assessing the association between SBP variability in a single visit and fatal and non-fatal cardiovascular events among the ELSA-Brasil participants in the context of the ongoing research project. The authors further suggest that future studies should be conducted, such as randomized clinical trials using different classes of antihypertensive drugs, in an attempt to determine the impact of these treatments on BPV, as well as establish associations with cardiovascular outcomes and mortality.

## Conclusion

The higher values of within-visit variability of SBP found in ELSA-Brasil participants at baseline were associated with higher cardiovascular risk, especially among males, regardless of mean SBP.
